# Haptoglobin Attenuates Cerebrospinal Fluid Hemoglobin-Induced Neurological Deterioration in Sheep

**DOI:** 10.1007/s12975-024-01254-9

**Published:** 2024-04-23

**Authors:** Bart R. Thomson, Nina Schwendinger, Katrin Beckmann, Thomas Gentinetta, Daniel Couto, Sandra Wymann, Valérie Verdon, Raphael M. Buzzi, Kevin Akeret, Peter W. Kronen, Eva M. Weinberger, Ulrike Held, Frauke Seehusen, Henning Richter, Dominik J. Schaer, Michael Hugelshofer

**Affiliations:** 1 Department of Neurosurgery, Clinical Neuroscience Center, Universitätsspital and University of Zurich, Zurich, Switzerland; 2 Division of Internal Medicine, Universitätsspital and University of Zurich, Zurich, Switzerland; 3 Neurology Service, Department of Small Animals, Vetsuisse Faculty, University of Zurich, Zurich, Switzerland; 4 CSL Biologics Research Centre, Swiss Institute for Translational and Entrepreneurial Medicine, CSL, sitem-insel, Bern, Switzerland; 5 Veterinary Anaesthesia Services – International, Winterthur, Switzerland; 6 Center for Applied Biotechnology and Molecular Medicine (CABMM), University of Zurich, Zurich, Switzerland; 7 Department of Biostatistics and Epidemiology, Biostatistics and Prevention Institute, University of Zurich, Zurich, Switzerland; 8 Laboratory of Animal Model Pathology, Institute of Veterinary Pathology, Vetsuisse Faculty, University of Zurich, Zurich, Switzerland; 9 Diagnostic Imaging Research Unit (DIRU), Clinic for Diagnostic Imaging, Vetsuisse Faculty, University of Zurich, Zurich, Switzerland

**Keywords:** Haptoglobin, CSF-Hb, Repeated exposure, Therapeutic benefit

## Abstract

Secondary brain injury (SBI) occurs with a lag of several days post-bleeding in patients with aneurysmal subarachnoid hemorrhage (aSAH) and is a strong contributor to mortality and long-term morbidity. aSAH-SBI coincides with cell-free hemoglobin (Hb) release into the cerebrospinal fluid. This temporal association and convincing pathophysiological concepts suggest that CSF-Hb could be a targetable trigger of SBI. However, sparse experimental evidence for Hb’s neurotoxicity in vivo defines a significant research gap for clinical translation. We modeled the CSF-Hb exposure observed in aSAH patients in conscious sheep, which allowed us to assess neurological functions in a gyrencephalic species. Twelve animals were randomly assigned for 3-day bi-daily intracerebroventricular (ICV) injections of either Hb or Hb combined with the high-affinity Hb scavenger protein haptoglobin (Hb-Hp, CSL888). Repeated CSF sampling confirmed clinically relevant CSF-Hb concentrations. This prolonged CSF-Hb exposure over 3 days resulted in disturbed movement activity, reduced food intake, and impaired observational neuroscores. The Hb-induced neurotoxic effects were significantly attenuated when Hb was administered with equimolar haptoglobin. Preterminal magnetic resonance imaging (MRI) showed no CSF-Hb-specific structural brain alterations. In both groups, histology demonstrated an inflammatory response and revealed enhanced perivascular histiocytic infiltrates in the Hb-Hp group, indicative of adaptive mechanisms. Heme exposure in CSF and iron deposition in the brain were comparable, suggesting comparable clearance efficiency of Hb and Hb-haptoglobin complexes from the intracranial compartment. We identified a neurological phenotype of CSF-Hb toxicity in conscious sheep, which is rather due to neurovascular dysfunction than structural brain injury. Haptoglobin was effective at attenuating CSF-Hb-induced neurological deterioration, supporting its therapeutic potential.

**Supplementary Information:**

The online version contains supplementary material available at 10.1007/s12975-024-01254-9.

## Introduction

The long-term functional outcome of patients with aneurysmal subarachnoid hemorrhage (aSAH) is strongly influenced by secondary brain injury (SAH-SBI), usually occurring between 4 and 14 days after the ictus [1]. Current clinical practice lacks causal treatment strategies to prevent SAH-SBI, which defines an unmet need for therapeutic innovation [1, 2].

Cell-free hemoglobin in cerebrospinal fluid (CSF-Hb) is suspected to be a driver for SAH-SBI [2–4]. In previous studies, we discovered that the delocalization of CSF-Hb into the brain’s interstitial spaces and the muscular layers of cerebral arteries initiates toxicity, which can be blocked by haptoglobin through a size-dependent mechanism [5]. By design, these studies were constrained by only measuring surrogate markers of physiological impairment instead of directly demonstrating that CSF-Hb leads to preventable functional neurological adverse effects [2, 5–7]. Therefore, evidence for the protective effects of haptoglobin against CSF-Hb-induced neurological deterioration in vivo is currently limited to mice [8]. The current study aimed to characterize the neurological phenotype of awake sheep during prolonged CSF-Hb exposure and to test the potential of haptoglobin to protect neurobehavioral functions in a large gyrencephalic animal model.

## Methods

We bi-daily administered purified Hb or Hb-haptoglobin (Hb-Hp) complexes via an EVD to conscious sheep for 3 consecutive days. The animals were allocated to the treatment group by block randomization (*n* = 6 per group). Before each compound administration, CSF was sampled for measurements of hemoprotein concentration and quantification of free and haptoglobin bound Hb by spectrophotometry and size exclusion chromatography (SEC). An implanted telemetry probe monitored movement, temperature, and intracranial pressure (ICP), while video recording tracked food intake and allowed for neurological scoring by a veterinary neurologist. After euthanasia, the brain was harvested. Preoperative CT and pre-terminal MRI scans were assessed for hydrocephalus, ischemia, or bleeding. The clinical readouts movement and food intake and a clinical neuroscore (defined in supplemental material) were analyzed using a generalized additive model (GAM) with non-linear spline fit for time (days). Repeated measurements over circadian rhythm were accounted for with random effects while additionally accounting for baseline differences. All investigators were blinded to treatment groups. Statistical analyses were performed in R. The authors complied with the ARRIVE guidelines.

## Results

### Quantification of CSF-Hb and Hb-Hp Complexes in CSF

**Fig. 1:**
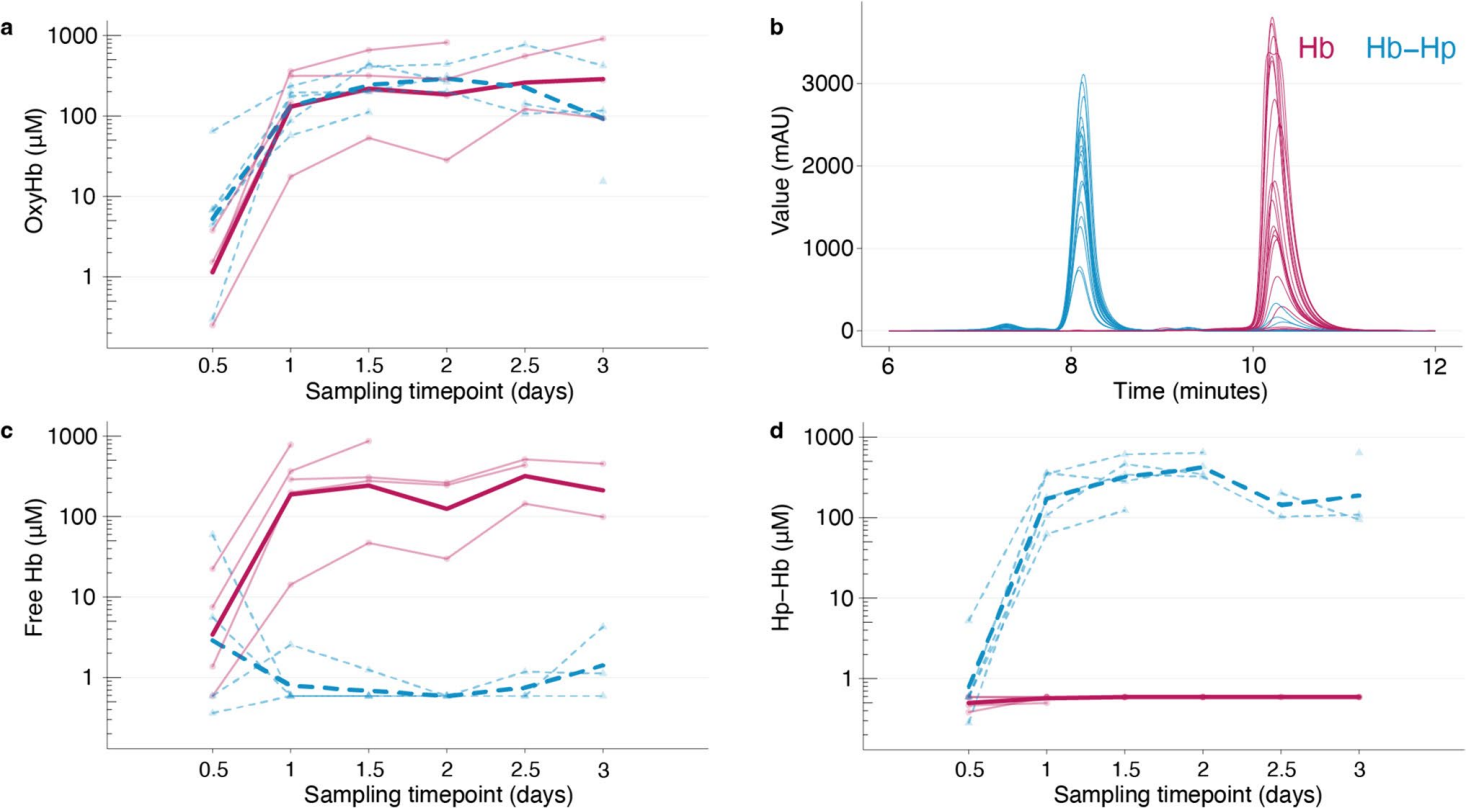
CSF-Hb spectrophotometry and size-exclusion chromatography of individual measurements. **a** OxyHb concentrations (spectrophotometry), **b** chromatograms of all sampling timepoints in all sheep, **c** free Hb, and **d** Hb-Hp complexes (SEC). Hb (red), Hb-Hp (blue), mean indicated in bold line

OxyHb exposure was consistently similar in both groups during the experiment (Fig. 1a). In the Hb-Hp group, SEC analysis revealed that most Hb remained complexed, indicating low free Hb concentrations (Fig. 1b). No relevant Hb signal was detected throughout the observation period in this group (Fig. 1c). Conversely, in the Hb-group, minimal Hb-Hp complexes formed, indicating the negligible endogenous haptoglobin concentrations in CSF (Fig. 1d).

### Movement and Food Intake

**Fig. 2:**
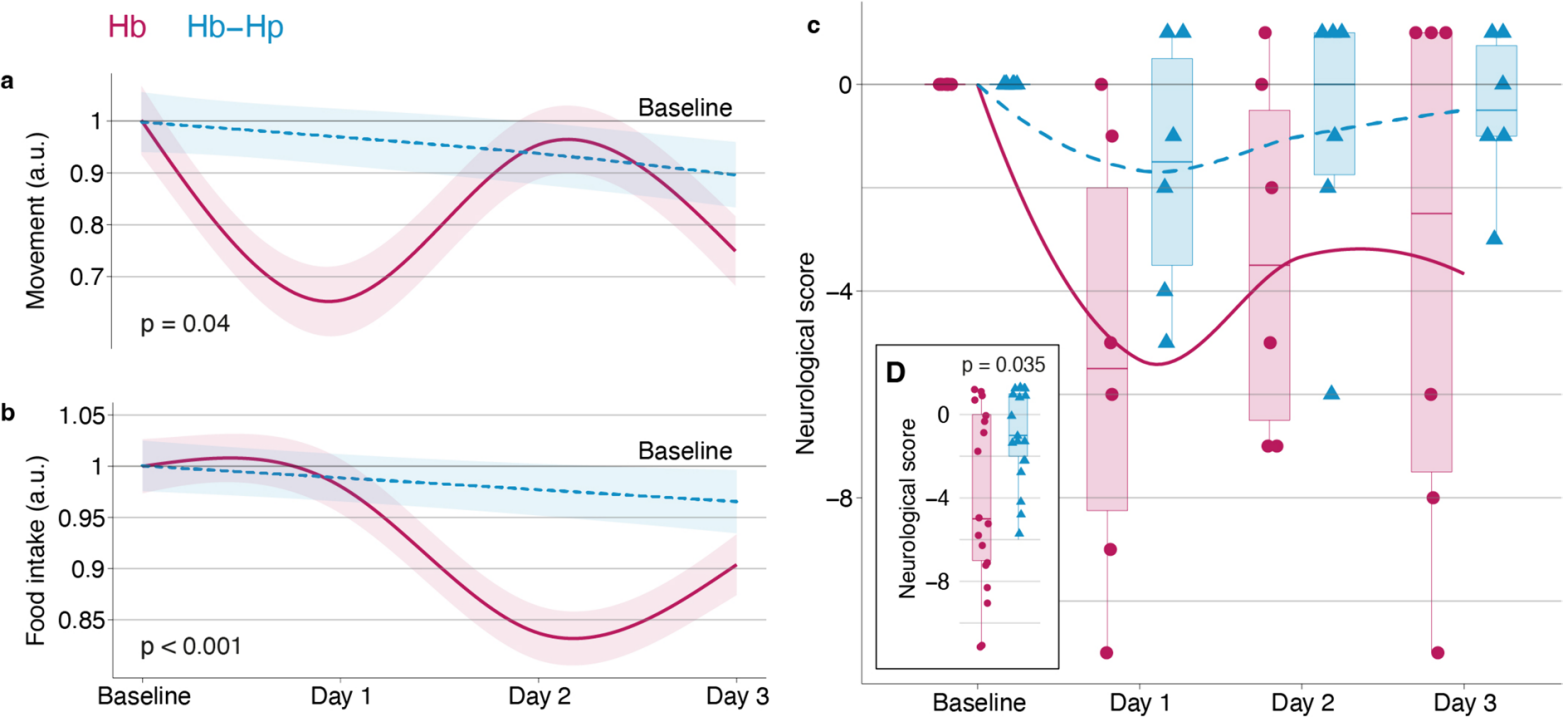
Temporal profile of clinical readouts. Non-linear course over time of **a** movement during wake hours (09:00–19:00) and **b** food intake fitted with a generalized additive model with y-axis scale in arbitrary units (a.u.). **c** Change in neurological observation score relative to the last day before treatment. **d** Overall change in clinical neuroscore over all treatment days in both groups. Hb (red), Hb-Hp (blue)

Hb treatment disturbed movement activity and food intake. These effects were attenuated by haptoglobin (activity: treatment effect coefficient = 0.12, SE = 0.06, *p* = 0.04, Fig. 2a, food intake: treatment effect coefficient = −0.06, SE = 0.004, *p* < 0.001, Fig. 2b). Movement activity and food intake have been analyzed using a GAM with a non-linear spline fit (4 knots) for time (day after the first injection), a random effect for circadian rhythm during individual days, and adjustment for differences in the baseline.

### Clinical Neuroscore

Following the first treatment day, a decrease in observational neuroscore was observed in both groups (Fig. 2c), partially recovering over the course of the experiment. The mean change in neuroscore over all treatment days was significantly lower in the Hb group compared to the Hb-Hp group according to a Wilcoxon rank sum test (*p* = 0.035, Fig. 2d).

### Histology and MR Imaging

Immunohistologically, Iba1-positive cell counts were comparable in both Hb and Hb-Hp-treated animals, while both treatment groups displayed a reduced vascular lumen area faction compared to controls, indicative for vasoconstriction (Supplemental Fig. 3). Pre-terminal MR imaging presented no significant group differences. Comprehensive details of these observations are presented in the supplemental material.

## Discussion

We demonstrated that CSF-Hb exposure leads to impaired neurological function in awake sheep. CSF-Hb led to disturbed movement activity, reduced food intake, and reduced neurological scoring. Co-administration of haptoglobin significantly attenuated the observed phenotype. In addition, we found no signals of Hp-induced adverse effects, such as epileptic seizures, hydrocephalus, or increased iron deposition in the brain. Collectively, these data support the safety and efficacy of haptoglobin supplementation as a potential therapeutic strategy to reduce CSF-Hb toxicity in aSAH patients.

In an observational clinical study, we detected CSF-Hb concentrations up to 200 µM, peaking around day ten post hemorrhage [3]. We aimed to model these clinically relevant exposure conditions. Importantly, complex formation with haptoglobin did not affect Hb concentrations in the CSF or overall iron deposition in the brain or perineuronal tissues. This implies similar clearance pathways and rates for cell-free Hb and its complexes with Hp, respectively. Immunohistochemistry for Iba1-positive macrophages delineated an enhanced macrophage accumulation in both Hb and Hb-Hp-treated animals compared to untreated controls, with a trend towards further enhancement in Hb-Hp over Hb-treated animals. These macrophages likely reflect a heme stress-induced adaptive mechanism [9].

The CSF-Hb-induced neurological impairment in our study is most likely caused by neurovascular dysfunction and not by structural injury, which is consistent with the absence of radiographic or histological differences between the treatment groups. A recent study showed that Hb exposure in neuronal cell cultures reduces AMPA-receptor-mediated synaptic currents and a downregulation of GluA1 at the postsynaptic membrane, leading to impaired neuronal electrical signaling capacity [7]. Even partial scavenging of Hb by haptoglobin below a certain threshold prevented neuronal dysfunction in these experiments. Moreover, we have shown in sheep that CSF-Hb leads to reduced cerebrovascular reactivity measured by blood oxygenation level-dependent functional MRI, which may impair neurovascular coupling [6]. The direct toxic impact of CSF-Hb on neuronal signaling and microvascular dysfunction may explain the clinical phenotype observed in our experiments. Therefore, structural vascular imaging modalities, e.g., digital subtraction angiography, may not be sufficient to monitor treatment effects of Hp in clinical trials.

Several limitations warrant attention. The sheep model focuses on CSF-Hb toxicity in the upper concentration range observed in aSAH patients. Still, it does not capture all factors influencing neurological outcomes, such as early brain injury or other blood components in CSF post-aSAH, such as activated coagulation factors or complement [2]. The 3-day study period, determined by ethical and animal welfare considerations, may not fully reflect the more prolonged CSF-Hb exposure in aSAH patients. This relatively brief exposure and the lack of peak regional Hb concentrations may explain the absence of overt cerebral ischemia in our experiments. Yet, it is expected that in the complete aSAH scenario, the adverse effects of CSF-Hb would be intensified through disease-propagating interactions with inflammation, coagulation, and other aSAH-related pathways [2]. The timing of haptoglobin administration may be critical for therapeutic efficacy in aSAH patients—our model did not provide a suitable platform to assess this potential temporal dependence reliably. Due to the altered CSF circulation after aSAH in patients, extensive pharmacokinetic data must be collected during early clinical trials with haptoglobin.

## Conclusion

Our study delineates an adverse neurological phenotype of CSF-Hb toxicity in a gyrencephalic species, which haptoglobin attenuates. There was no evidence for additional toxicity caused by Hb-Hp complexes, excessive iron accumulation, or disturbance of CSF circulation. These data support translational efforts in developing haptoglobin-based therapeutics to prevent CSF-Hb-induced toxicity in aSAH patients.

## Electronic Supplementary Material

Below is the link to the electronic supplementary material.

Supplementary Material 1

Supplementary Material 2

Supplementary Material 3

## Data Availability

The data that support the findings of this study are available from the corresponding author upon reasonable request. Data are located in controlled access data storage at University of Zurich, Switzerland.
