# Supplementary material for: Haptoglobin Attenuates Cerebrospinal Fluid Hemoglobin-Induced Neurological Deterioration in Sheep
Source: Transl Stroke Res. 2024 Apr 23;16(3):728–32. doi: 10.1007/s12975-024-01254-9 (PMC12045829; doi:10.1007/s12975-024-01254-9)
Supplement: Supplementary file 1 — Supplementary Material 1 [file 12975_2024_1254_MOESM1_ESM.pdf]

Supplemental Tables

Supplemental Table 1. Sheep neurological health evaluation matrix. Description of grading parameters for alertness, gait, posture, appetite, respiration, and pain response.

Score	Alertness	Gait	Posture	Appetite	Respiration	Pain
1	Not responsive, apathetic	Severely abnormal or no weight bearing	Lying, not standing even when forced; great difficulties	Not eating	Severely abnormal breathing pattern/frequency, strong pumping	Severe pain: constant teeth grinding, hanging ears, frequent flehming, partial/complete eyelid closure, inappetence
2	Responsive, but not interested	Moderately to severely abnormal weight bearing/gait	Lying, but stands when forced; unstable and immobile	Eating hesitantly and discontinuously	Moderate to severe abnormal breathing pattern/frequency and pumping	Moderate/severe pain: frequent teeth grinding, flehming, reaction to slight touch at wound area
3	Less interested in environment	Moderately abnormal weight bearing/gait	Lying, but stands immediately; unstable, moves slowly	Moderately reduced appetite	Moderately abnormal breathing pattern/frequency	Moderate pain: intermittent teeth grinding, partial eyelid closure, occasional flehming, reaction to pressure at wound area
4	Slightly less interested in environment	Slightly abnormal weight bearing/gait	Lying more often than normal, moves slowly	Slightly reduced appetite	Slightly abnormal breathing pattern/frequency	Mild pain: slight signs of discomfort, slight reaction to pressure at wound area
5	Normal, interested in environment	Normal weight bearing/gait	Normal physiological posture	Normal appetite	Normal breathing pattern/frequency	No pain, normal general condition
