# Supplementary material for: Haptoglobin Attenuates Cerebrospinal Fluid Hemoglobin-Induced Neurological Deterioration in Sheep
Source: Transl Stroke Res. 2024 Apr 23;16(3):728–32. doi: 10.1007/s12975-024-01254-9 (PMC12045829; doi:10.1007/s12975-024-01254-9)
Supplement: Supplementary file 2 — Supplementary Material 2 [file 12975_2024_1254_MOESM2_ESM.pdf]

Supplemental Figures

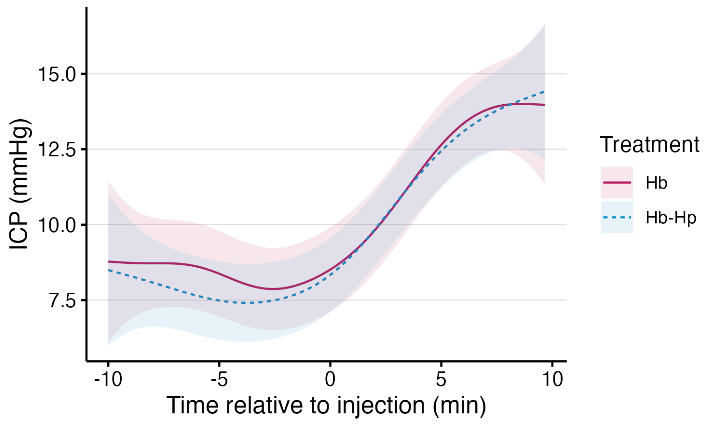

Supplemental Figure 1. Injection control and ventricular volume evaluation. Changes in ICP during the first injection timepoint in both groups.

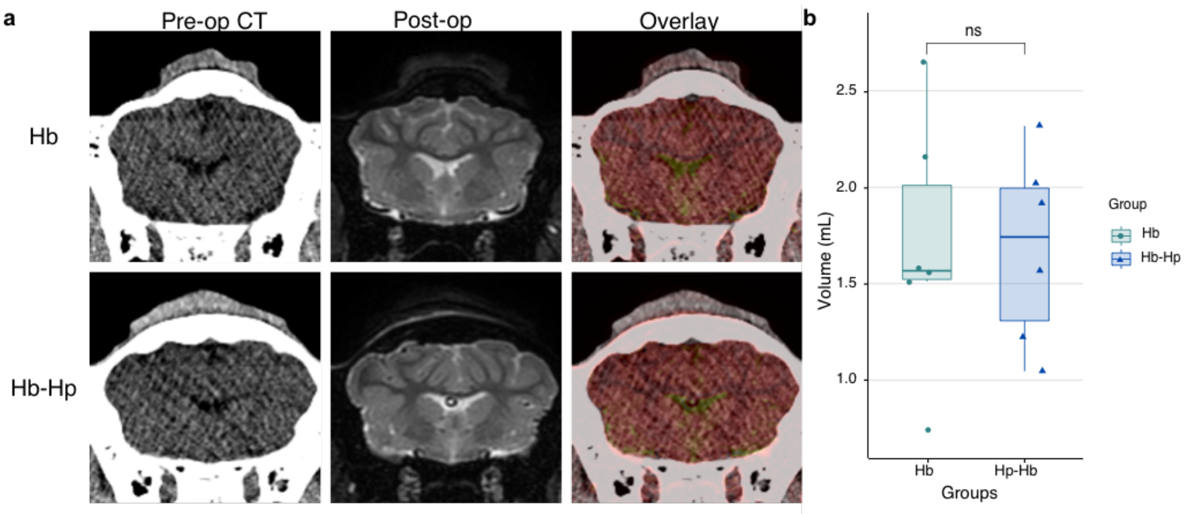

Supplemental Figure 2. Visual comparison of ventricular imaging in representative animals. (**a**) Registered pre- (CT, left column) and post-operative (T2, middle column) imaging and both overlaid (right column) of the lateral ventricles. (**b**) Volumetric comparison of ventricular CSF on the post-operative T2.

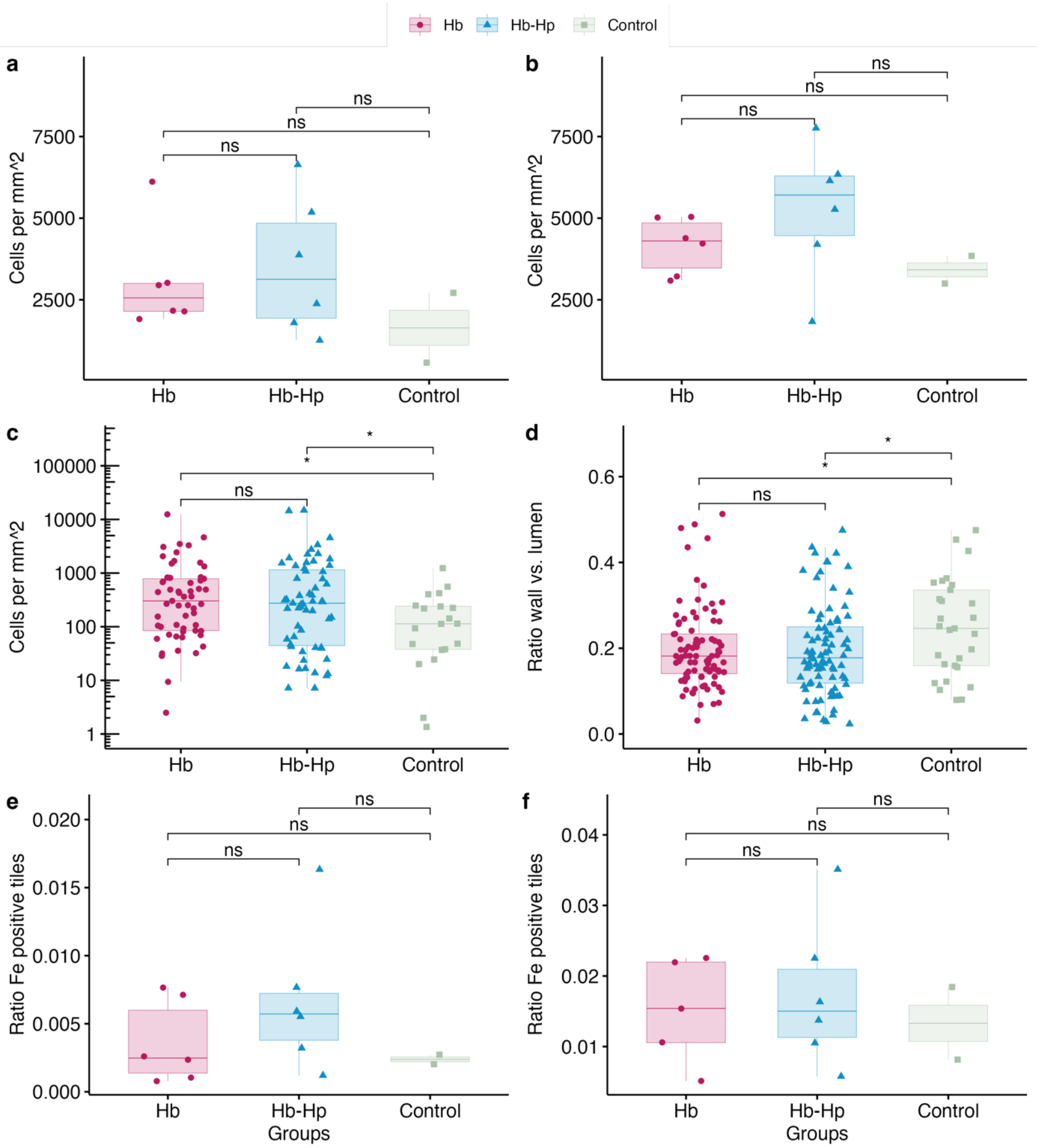

Supplemental Figure 3. Histological stainings. **(a)** Inflammatory cells stained by Iba1 in a standardized anatomical region of interest and in **(b)** the periventricular area. Smooth muscle cells stained by SMA indicating the **(c)** positively stained cells in the perivascular cuff and the **(d)** surface ratio of arteriolar wall in comparison to arteriolar lumen. DAB-enhanced iron staining of 10x10 µm tiles, highlighting the ratio of positive cells in **(e)** the parenchyma and **(f)** the periventricular area.
