# Supplementary material for: Haptoglobin Attenuates Cerebrospinal Fluid Hemoglobin-Induced Neurological Deterioration in Sheep
Source: Transl Stroke Res. 2024 Apr 23;16(3):728–32. doi: 10.1007/s12975-024-01254-9 (PMC12045829; doi:10.1007/s12975-024-01254-9)
Supplement: Supplementary file 3 — Supplementary Material 3 [file 12975_2024_1254_MOESM3_ESM.pdf]

Supplemental Material

Methods

Animals and procedure

This study was conducted according to the Swiss Animal Welfare Act (TschG, 2005) and the Swiss Animal Welfare Ordinance (TSchV, 2008) received ethical approval from the Swiss Federal Veterinary Office Zurich (animal license no. ZH171/2020). Twelve female Swiss alpine sheep (Staffelegghof, Küttigen bei Aarau, Switzerland) were chosen for this study with a median weight of 90 kg (weight range: 76.5 – 103 kg) and an age between 2-4 years. Prior to surgery, they were allowed to acclimatize to their new environment for at least 7 days. A veterinarian performed standardized screening by means of physical examination and blood testing. Prior to surgery the animals were fasted with free access to water for 18-24 hours.

Anaesthesia

Standardized anesthesia for surgical and preterminal imaging procedures was performed, cardiovascular and respiratory health checks preceded sedation and anesthesia induction. Following an 18-24 hour fasting period, animals received premedication with buprenorphine (0.01 mg/kg BW, IM) and medetomidine (0.007-0.01 mg/kg BW), along with prophylactic antibiotics (penicillin 30,000 IU/kg BW and gentamicin 4 mg/kg BW, IV) and pre-emptive analgesic carprofen (4 mg/kg BW, IV). A tetanus booster (3,000 IU/sheep) was administered subcutaneously.

Anesthesia induction involved midazolam (0.1 mg/kg BW, IV), ketamine (3-5 mg/kg BW, IV), and propofol (0.4-4 mg/kg BW, IV), with laryngeal desensitization via lidocaine spray and tracheal intubation confirmed by FetCO2 monitoring. Maintenance was achieved with isoflurane (1%-3%) in oxygen, propofol (0.5-1 mg/kg/h), and ketamine (20-50μg/kg/h) infusions. Corneal protection was provided by ophthalmic ointment.

Intraoperatively, Ringer's lactate was administered (5-10 mL/kg/h). Monitoring parameters included: ECG, heart rate, pulse rate, and invasive blood pressure measurements via an auricular arterial catheter. Monitoring also covered inspired and expired carbon dioxide, oxygen, isoflurane levels, esophageal temperature, and arterial blood saturation (SpO2), with parameters recorded every 10 minutes.

Surgery

Surgical navigation was used to implant an external ventricular drain (EVD, DePuy Synthes) into the lateral ventricle. Kink-free securing of the EVD was ensured using Ventrifix (Neuromedex GmbH, Hamburg, Germany). Following EVD implantation, a telemetry probe (Stellar Telemetry - Wireless Monitoring, TSE Systems) was subcutaneously implanted. This probe continuously measured intracranial pressure (ICP), temperature, and the sheep's general activity, capturing acceleration in the x, y, and z directions. While the animals were under general anesthesia, a skin incision was made in the neck to create a subcutaneous pouch using blunt dissection, in which the telemetry probe was inserted. A postoperative CT scan was performed to confirm correct EVD as well as ICP probe placement and to exclude surgical complications (e.g. significant intracranial bleeding).

Following the postoperative CT scan, the animals were given two full days for recovery. The treatment period started at day 3 post-op, during which cerebrospinal fluid (CSF) sampling and substance infusion were conducted twice daily via sterile connection to the EVD. If a sample contained less than 200 µL of CSF, it was excluded from further analysis as this indicated poor sample quality, due to the dead space volume of the catheter. After CSF sampling, 1 mL of 3 mM of the respective substance was infused at a rate of 60 mL/h using a syringe pump (PHD Ultra, Harvard Apparatus). To maintain uniform conditions, both groups underwent treatment in pairs, with group allocation within each pair determined by predefined randomization prior to the study. During the treatment period, ICP was routinely

monitored non-invasively via the implanted telemetric pressure sensor. To prevent clogging, the EVD was flushed with 1 mL of Ringer's solution at 60 mL/h immediately following each substance infusion.

On the fourth day, pre-terminal anatomical (T1, T1Gd, T2) and DTI MRI scans were conducted under general anesthesia conditions prior to euthanasia. The pre-terminal anatomical MRI scans served to evaluate hydrocephalus and facilitate volumetric comparisons between the two treatment groups. Additionally, the pre-terminal MRI aimed to identify other radiological findings such as ischemia or intracranial bleeding. Following euthanasia, death was confirmed by the absence of heartbeat and pupillary reflex after administering pentobarbital intravenously at a dosage of 100-150 mg/kg BW, and a volume of 0.3-0.5 ml/kg. Subsequently, the brain was harvested and prepared for histological analyses.

Hemoglobin, Haptoglobin and Haptoglobin-Hb complexes

Hemoglobin (Hb) was isolated from sheep blood using previously described methods [1]. Its concentrations were quantified via spectrophotometry. Concentrations are expressed in terms of total heme molarity, where 1 M Hb tetramer is equivalent to 4 M heme [2]. Haptoglobin was sourced from human plasma pools and its identity, purity, and functionality were authenticated through liquid chromatography (SEC-HPLC), PAGE, and BLI, according to established protocols [3–5].

To generate Hb-haptoglobin (Hb-Hp) complexes, 13.5 mM (expressed in heme equivalent) of purified sheep Hb was mixed with 3.4 mM of haptoglobin. These mixtures were then diluted with NaCl to achieve a concentration of 3 mM for the Hb-Hp complex. An excess of 10% haptoglobin ensured complete elimination of cell-free Hb. Spectrophotometry confirmed the correct concentrations.

Further validation for the absence of cell-free Hb was conducted using HPLC. Specifically, the complexes were separated using an analytical BioSep-SEC-s3000 (600 × 7.8 mm) LC column coupled with a BioSep-SEC-s3000 (75 × 7.8 mm) Guard Column (Phenomenex, Torrance, CA). These were connected to an LKB 2150 HPLC Pump (LKB-Produkter AB, Bromma, Sweden). A 20 mM potassium phosphate solution at pH 6.8 served as the mobile phase, with a flow rate of 1 mL/min. Absorption was monitored at 414 nm using a Jasco UV-970 Intelligent UV/VIS Detector (JASCO International Co., Ltd., Tokyo, Japan) and recorded with Lab Chart software version 7.2.1 (AD Instruments, Hastings, UK). Visual inspection confirmed the absence of cell-free Hb in the samples.

Analysis of CSF-Hb and Heme Metabolites

Spectrophotometry experiments were conducted in 96 well-plates at room temperature using a Lunatic spectrophotometer (Unchained Labs, USA). The absorbance of the samples was measured in the 350 to 650 nm range. A spectral deconvolution script was used for quantification of oxyHb in CSF. This involved fitting reference spectra of known oxyHb concentrations to the measured spectra, employing a nonnegative least squares algorithm as previously described[2].

Further analysis of sheep CSF samples was carried out using SEC–high-performance liquid chromatography on an Agilent 1260 II HPLC, equipped with a quaternary pump and a photodiode array detector (DAD) from Agilent. These samples, along with hemoglobin standards, were separated on a Diol-300 column (3 µm, 300 × 8.0 mm, YMC Co. Ltd.), utilizing PBS (pH 7.4, Bichsel) as the mobile phase, maintaining a flow rate of 1 mL/min. Dual wavelengths (λ = 280 nm and λ = 414 nm) were recorded for every sample. From this information, peak areas for Hb-Hp complexes, free hemoglobin, and other proteins bonded with heme were quantified.

Clinical parameters

Throughout the treatment phase, intracranial pressure (ICP) was continuously monitored to gauge potential intracranial hypertension induced by intraventricular injection (hydrocephalus), hemorrhagic complications or brain oedema. During each substance injection, ICP was observed as confirmation of successful intracranial administration. Body temperature measurement served as an indicator for postsurgical recovery, vegetative dysregulation, and possible infection. Movement data, recorded at a frequency of 1 Hz in 3-axes, was exported using Notocord-hem software (NOTOCORD Systems, Croissy-sur-Seine, France). Prior to any further analysis, the data was downsampled to 1/120 Hz, to reduce granularity and focus on long-term effects.

More complex animal behavior and food intake were observed through two high-resolution cameras per sheep (1280x720 resolution, Bascom Bullet, Düsseldorf, Germany). One of the cameras was angled towards the sheep's feeding bin, enabling automated content segmentation. Ground truth delineations for random video frames were initially provided by the Segment Anything model [6]. After manual removal of incorrect segmentations, a refined dataset consisting of 3973 frames was created (2579 train, 778 validation, 616 test), which were then used to train the final YOLOv8 model [7].

A board-certified veterinary neurologist conducted neurological scoring based on the video data to evaluate the health and well-being of the sheep in this study. To ensure objectivity and reliability, the assessment was performed fully blinded to treatment allocation. Five consecutive hours of undisturbed video footage, collected on the last day of the recovery period as well as throughout all three treatment days were used for scoring. This prolonged observation period allowed for capturing behavioral and conditional variances, providing a comprehensive view of the sheep's neurological and physiological states. The animals were scored on alertness, posture, appetite, respiratory rate, pain, and weight bearing (Supplemental Table 1). Neurobehavioral scores were analyzed with a student t-test.

Histology

Consecutive tissue sections (3-5 µm) were stained with hematoxylin and eosin (HE), iron stain enhanced with 3,3′-diaminobenzidine (DAB), or immunohistochemistry (IHC). For IHC, the horseradish peroxidase (HRP) method was employed to identify macrophages/activated microglial cells (Iba1+), smooth muscle antigen (SMA+).
Sections were first deparaffinized and then subjected to antigen retrieval using citrate buffer (pH 6.0) or Tris/EDTA buffer (pH 9.0) for 20 minutes at 98 °C, followed by incubation with the primary antibodies, which were diluted in a specialized dilution buffer (Agilent Dako, Glastrup, Denmark). Next, endogenous peroxidase was blocked (peroxidase block, Agilent Dako, Glastrup, Denmark) for 10 minutes at room temperature. The sections were then incubated with the appropriate secondary antibodies or detection systems using an autostainer (either Dako Agilent or Ventana). A final counterstaining was performed with hematoxylin. As a quality control measure, brain sections served as internal positive controls for Iba1 and SMA antigen expression. Sections that were incubated without primary antibodies acted as negative controls.

QuPath v0.4.3 was used to quantify histology, and detailed analysis was performed in Python. In order to quantify the Iba1+ cells, two regions of interest were defined, to which cell counts were standardized. One region being a 500µm^2 frontal cortical region, the second region a 150µm wide periventricular band.

As a marker of adaptive/inflammatory response the number of macrophages within the perivascular cuff was quantified and standardized to the area of the cuff as well as the vessel lumen. Ten vessels were visualized by a SMA staining. The fraction of the inner lumen in relation to the outer lumen was used as a marker for vasoconstriction (lumen area fraction). 15 parenchymal vessels were delineated manually per animal.

The amount of iron was quantified by defining the same regions of interest as for the Iba1. The areas of interest were divided into 5μm^2 tiles, and a neural network was trained to detect the DAB-enhanced iron staining. The tiles were dichotomized as positive or negative detections by the algorithm. The ratio of positive to negative detection was standardized to the areas of interest.

Statistical analysis

Statistical analyses were conducted using R statistical software, version 3.6.3[8]. For categorical variables, descriptive statistics are displayed as absolute numbers (n) and percentages (%). Continuous variables are presented as the mean along with the standard deviation (SD).

Results

Exclusion of animals

Due to removal of their own catheters, two (paired) sheep could not be infused with a substance during the treatment period. Due to inclusion in the study, they were used as control animals in the histological analysis. As a result, total group numbers were: 6 Hb sheep, 6 Hb-Hp sheep and 2 control sheep.

ICP and temperature

Similar to movement activity and food intake, a generalized additive model (GAM) with non-linear spline fit for time relative to first injection was used to model ICP (mmHg) for both groups. Due to the high variability of ICP depending on head position with respect to the body, only the 20 minutes surrounding the injection are shown (-10 minutes - + 10 minutes). There was no evidence for a difference in ICP increase between the two groups over time (treatment effect coefficient = -0.30, SE = 0.50, p = 0.54, *Supplemental Figure 1*). More

detailed comparison of ICP differences between groups over the whole treatment period was not feasible due to technical limitations and data variability. However, no qualitative group differences could be observed.

Qualitative analysis of body temperature did not reveal obvious differences between groups. Initial increase of body temperature at day one reflects post-surgical recovery period with a plateau reached at day 2. No evidence was found for induced vegetative dysregulation or fever in any of the animals.

MR Imaging

Pre-terminal anatomical (T1, T1Gd, T2) and DTI MRI sequences were performed in all animals. No obvious differences in radiological findings between groups were observed. Variable extent of perifocal edema along the parenchymal EVD-trajectory was observed without obvious differences between the two groups (*Supplemental Figure 2a*). A variable degree of cerebral edema was detected surrounding the EVD trajectory without a clear pattern based on treatment group. No major ischemic infarction or intraparenchymal hemorrhage was detected. In a comparison of the pre-surgical CT scans with the pre-terminal MRI scans for each animal, no evidence to suggest the induction of significant hydrocephalus was found. Volumetry of the supratentorial ventricular system did not show a significant difference (independent t-test) between treatment groups ($p = 0.96$, *Supplemental Figure 2b*).

Histology

No significant group differences in cell count were observed in the Iba1 staining, neither in the standardized cortical region (Hb vs. Hb-Hp, $p=0.67$; Hb vs. Control, $p=0.39$; Hb-Hp vs. Control, $p=0.28$, *Supplemental Figure 3a*), nor in the periventricular area (Hb vs. Hb-Hp, $p=0.27$; Hb vs. Control, $p=0.28$; Hb-Hp vs. Control, $p=0.10$, *Supplemental Figure 3b*).

In the SMA staining, an increase in cell numbers was observed in animals treated with either Hb or Hb-Hp in the ventricular cuff, compared to the control group (Hb vs. Hb-Hp, p=0.56; Hb vs. Control, p=0.01; Hb-Hp vs. Control, p=0.01, *Supplemental Figure 3c*). Moreover, a significantly smaller lumen area fraction was observed (indicating vasoconstriction) for both treatment groups when compared to the control group (Hb vs. Hb-Hp, p=0.76; Hb vs. Control, p=0.04; Hb-Hp vs. Control, p=0.03, *Supplemental Figure 3c*).

In the DAB-enhanced iron staining, no significant differences were found within the parenchyma (Hb vs. Hb-Hp, p=0.25; Hb vs. Control, p=0.38; Hb-Hp vs. Control, p=0.11, *Supplemental Figure 3e*) or the periventricular area (Hb vs. Hb-Hp, p=0.69; Hb vs. Control, p=0.79; Hb-Hp vs. Control, p=0.60, *Supplemental Figure 3f*).

References

1. Elmer, J., Harris, D. R., Sun, G. & Palmer, A. F. Purification of hemoglobin by tangential flow filtration with diafiltration. *Biotechnol. Prog.* **25**, 1402–1410 (2009).
2. Deuel, J. W. *et al.* Different target specificities of haptoglobin and hemopexin define a sequential protection system against vascular hemoglobin toxicity. *Free Radic. Biol. Med.* **89**, 931–943 (2015).
3. Lipiski, M., Deuel, J. W. & Baek, J. H. Human Hp1-1 and Hp2-2 Phenotype-Specific Haptoglobin Therapeutics Are Both Effective In Vitro and in Guinea Pigs to Attenuate Hemoglobin Toxicity. *Antioxid. Redox Signal.* (2013).
4. Pimenova, T., Pereira, C. P., Schaer, D. J. & Zenobi, R. Characterization of high molecular weight multimeric states of human haptoglobin and hemoglobin-based oxygen carriers by high-mass MALDI MS. *J. Sep. Sci.* **32**, 1224–1230 (2009).
5. Buzzi, R. M. *et al.* Modular Platform for the Development of Recombinant Hemoglobin Scavenger Biotherapeutics. *Mol. Pharm.* **18**, 3158–3170 (2021).
6. Kirillov, A. *et al.* Segment Anything. *arXiv [cs.CV]* (2023).
7. Jocher, G., Chaurasia, A. & Qiu, J. YOLO by Ultralytics (Version 8.0. 0)[Computer software]. (2023).
8. *R: A Language and Environment for Statistical Computing : Reference Index*. (R Foundation for Statistical Computing, 2010).
